# Is the Current Screening Availability for Early Stages of Type 1 Diabetes in Germany Related to the Population‐Based Frequency of Diabetic Ketoacidosis at Clinical Manifestation in Children and Adolescents

**DOI:** 10.1155/pedi/6905472

**Published:** 2026-02-08

**Authors:** Alexander J. Eckert, Joachim Rosenbauer, Clemens Kamrath, Marina Sindichakis, Ansgar Thimm, Martin Holder, Jantje Weiskorn, Daniel P. Lorenz, Susanne Gonzalves, Gita Gemulla, Sven Golembowski, Ute Ohlenschläger, Dieter Hüseman, Katja Palm, Andreas Lemmer, Valentina Lahn, Antonia Müller, Donald Wurm, Marjatta Wütherich, Reinhard W. Holl, for the DPV-Wiss-Initiative

**Affiliations:** ^1^ Institute of Epidemiology and Medical Biometry, University of Ulm, Ulm, Germany, uni-ulm.de; ^2^ German Center for Diabetes Research (DZD), Munich-Neuherberg, Germany, dzd-ev.de; ^3^ Institute for Biometrics and Epidemiology, German Diabetes Center (DDZ), Leibniz Center for Diabetes Research at Heinrich Heine University Düsseldorf, Düsseldorf, Germany, uni-duesseldorf.de; ^4^ Department of General Pediatrics, Division of Pediatric Endocrinology and Diabetology, Center of Child and Adolescent Medicine, University of Freiburg, Freiburg, Germany, uni-freiburg.de; ^5^ Hospital for Children and Adolescents, Klinikum Traunstein, Traunstein, Germany; ^6^ Department of General Pediatrics, Sana-Hospital Remscheid, Remscheid, Germany, sana.de; ^7^ Department of Pediatric Endocrinology and Diabetology, Olgahospital, Klinikum Stuttgart, Stuttgart, Germany, klinikum-stuttgart.de; ^8^ Diabetes Center for Children and Adolescents, Auf der Bult, Hannover, Germany, auf-der-bult.de; ^9^ Clinic for Child and Adolescent Medicine, Klinikum Frankfurt Höchst, Frankfurt, Germany, klinikumfrankfurt.de; ^10^ Department of Pediatrics, Diakonissen-Stiftungs-Krankenhaus, Speyer, Germany, diakonissen.de; ^11^ Department of Pediatrics, Faculty of Medicine and University Hospital Carl Gustav Carus, Technische Universität Dresden, Dresden, Germany, tu-dresden.de; ^12^ Department of Pediatrics, Sana Hospital Lichtenberg, Berlin, Germany, sana.de; ^13^ Pediatrics, Endocrinology and Diabetology, Friedrich-Ebert Krankenhaus Neumünster, Neumünster, Germany, friedrich-ebert-krankenhaus.de; ^14^ Department of Pediatrics, Werner-Forßmann-Krankenhaus, Eberswalde, Germany; ^15^ Division of Endocrinology, Diabetology and Metabolic Medicine, University Children’s Hospital, Magdeburg, Germany, uni-magdeburg.de; ^16^ Department of Pediatrics and Adolescent Medicine, Helios Clinical Center, Erfurt, Germany, helios-kliniken.de; ^17^ Children’s Hospital Hamburg-Altona, Hamburg, Germany; ^18^ Clinic Group Dr. Guth GmbH and Co. KG, Clinical Center Karlsburg, Greifswalder Straße 11, Karlsburg, Germany; ^19^ Department of Paediatrics, Marienhaus Klinik Neunkirchen, Neunkirchen, Germany, marienhaus-klinikum.de; ^20^ Child and adolescent medicine, Klinikum Bremen-Nord, Bremen, Germany

**Keywords:** Fr1da, immigrant background, pediatric, physician density, prevention, urbanity index

## Abstract

**Aims:**

Is the current screening for early‐stage type 1 diabetes (T1D) associated with the rate of diabetic ketoacidosis (DKA) at T1D manifestation at population level?

**Materials and Methods:**

Children with T1D manifestation in 2015–2023 in Germany, aged 0.5 to <15 years from the multicenter diabetes registry (DPV) were included and allocated to federal states (FSs) based on their residential postal code. The relative risk (RR) with 95% confidence interval for DKA at manifestation of T1D by FS with vs. without screening, and demographic/context factors was calculated using logistic regression models.

**Results:**

24,408 children (54.3% males) were included with median onset age of 8.8 (quartiles: 5.3; 11.8) years. The RR for DKA was not significantly lower in FS with vs. without screening (RR: 0.96 [0.93–1.03], *p* = 0.393) overall, but in the sub‐group of children with the highest probability of being screened (age 1.75–10.99 years, manifestation in Bavaria from 2020 to 2023, RR: 0.88 [0.80–0.97], *p* = 0.012). The most important predictor for DKA was manifestation age <3 years (RR: 1.83, [1.66–2.03], *p* < 0.001).

**Conclusions:**

Until 2023, current regional screening initiatives for early‐stage T1D were not associated with lower frequency of DKA at population level, but might be helpful in the future if the coverage can be markedly increased.

**Summary**

• Until the end of 2023, the offer of a study participation for early stages of type 1 diabetes (T1D) was not associated with a decrease in diabetic ketoacidosis (DKA) proportion at pediatric T1D manifestation at the population level in Germany.
• A reduced prevalence of DKA may be demonstrated in the future with higher and more homogeneous participation throughout the federal states (FSs).
• Children with T1D onset before 3 years of age are at the highest risk for DKA at manifestation, but are difficult to capture with screening.
• Immigrant background and rural place of living are potentially amenable predictors of DKA at manifestation. No association with physician density was observed.

## 1. Introduction

Diabetic ketoacidosis (DKA) is a common and severe complication at clinical manifestation of type 1 diabetes (T1D). In Germany, about one in four children under the age of 18 years suffered from DKA at T1D onset between 2011 and mid‐2023. Furthermore the incidence of pediatric T1D with DKA was increasing [1]. Other European countries report DKA proportions at pediatric T1D manifestation between 20% and 40% [2] with even higher rates reported in the US [3]. The reasons for the high proportion of DKA at pediatric T1D manifestation and the recently reported increase in many countries even before the COVID‐19 pandemic are not fully understood. An important cause is the delayed diagnosis of diabetes due to failure to recognize or misdiagnosis of the typical symptoms of diabetes [4].

One attempt to lower the rate of DKA at T1D onset in children is to screen for early stages of T1D in order to raise awareness in children and their parents, and initiate insulin therapy early at clinical manifestation of the disease. Italy was one of the first European countries to implement a screening program for autoantibodies against T1D and celiac disease by law [5]. In Germany, the Institute for Diabetes Research at Helmholtz Munich offers an antibody‐based screening study since 2015 (Fr1da) for early detection of T1D as part of the routine pediatric checkup or during any visit. Between 2015 and 2021, children between the ages of 2 and 6 were offered participation in the study and, from 2021, children between the ages of 2 and 10. The Fr1da screening study was initiated in Munich, Bavaria, and later expanded to the whole federal state (FS) of Bavaria as well as to the FSs of Lower Saxony (10/2016), Hamburg (06/2021), and Saxony (2021) [6–8].

It was reported that the DKA proportion among participants of the Fr1da initiative who developed stage 3 T1D was very low (2.5%) compared to the above mentioned DKA rates [9]. However, the impact on the whole pediatric population is unclear. It must also be taken into account that the number of pediatricians who could participate in the Fr1da study varies from region to region. For example, the coverage of pediatricians (calculated as number of pediatricians compared to the number of required pediatricians) in Bavaria is 144% while in Lower Saxony it is 138% and in Hamburg it is the lowest in Germany with 120%. Otherwise in Saxony, the coverage of pediatricians is higher with 190% [10].

Some FSs, such as Thuringia, were part of programs for early detection of T1D based on genetic risk for T1D as part of the newborn screening (Freder1k‐study) [8] as a different approach. In Stuttgart, the state capital of the FS of Baden‐Württemberg, an awareness campaign to inform children and parents in schools and other facilities and sensitize healthcare providers for diabetes symptoms was implemented as another approach from 2015 to 2017 [11, 12]. This campaign was expanded from July 2021 to the FS of Baden‐Württemberg and in a slightly different version to a nationwide prevention campaign. However, the coverage rate of such prevention campaigns, and therefore its effectiveness for DKA prevention at the population level, is even more difficult to capture.

The aim of this study was to use a nationwide diabetes registry to provide evidence whether the current screening study Fr1da for early stages of T1D may be associated with the frequency of DKA at T1D onset of the whole pediatric population in Germany.

## 2. Materials and Methods

### 2.1. Data Collection

This analysis was based on data from the prospective, multicenter diabetes patient follow‐up registry (DPV). Developed as a standardized electronic health record at the Institute of Epidemiology and Medical Biometry, Ulm University, Germany, the DPV registry provides ongoing real‐world data on diabetes treatment and outcome from more than 500 centers in Germany, Austria, Switzerland, and Luxembourg since 1995. The coverage rate for children and adolescents with diabetes is estimated to be >90% for Germany [13]. The DPV initiative and the analysis of pseudonymized data were approved by the Ethics Committee of Ulm University (Approval Number: 314/21) as well as by local review boards. The data are transmitted twice a year, checked for inconsistency or implausibility, and reported back to the respective centers for correction, if necessary.

### 2.2. Design and Participants

**Figure 1:**
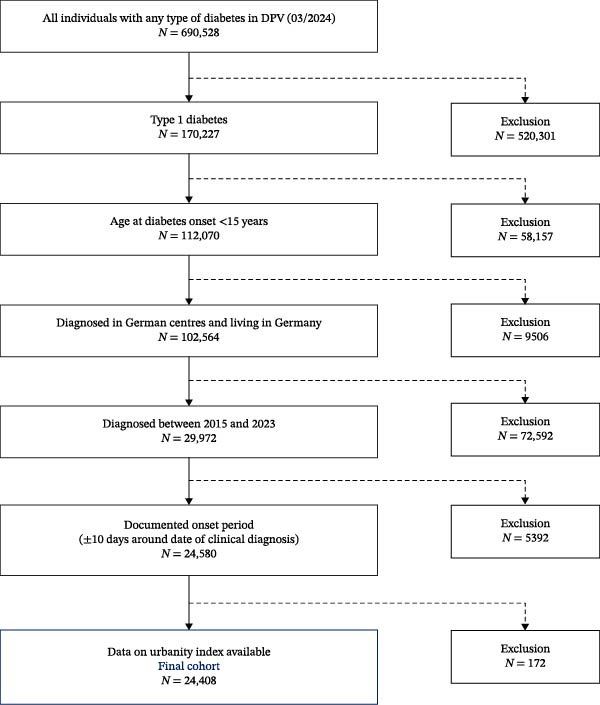
Flowchart of inclusion/exclusion criteria.

Children and adolescents documented in the DPV registry with newly diagnosed T1D from 2015 to 2023 in Germany and with age at onset <15 years were included (Figure 1). The Fr1da‐study provides the opportunity for autoantibody screening for T1D as part of routine pediatric checkups in selected regions (Bavaria, Saxony, Lower Saxony, and Hamburg during our observation period). Due to its voluntarily research‐study design, the study is not comprehensive. Thus, pediatricians play a major role for families to participation in the screening program. All of the other 12 FSs of Germany were considered as FSs with no screening possibility. Individuals were allocated to a FS by the postal code of their residence at time of manifestation. Since most of the screened children to date are from Bavaria, for sensitivity analysis, Bavaria was considered as the only FS with screening, therefore, excluding Hamburg, Lower Saxony, and Saxony from this analysis.

### 2.3. Data Management and Variable Definition

The diagnosis and classification of diabetes types was conducted by the diabetologists at the participating centers according to the clinical presentation of people with diabetes manifestation and lab results, based on German guidelines, which are identical to guidelines of the International Society for Pediatric and Adolescent Diabetes (ISPAD). DKA at manifestation was defined as pH‐value <7.3 and/or bicarbonate level <15 mmol/L, measured within 10 days of the date of diagnosis (ISPAD guideline, valid until 2021) [14]. HbA1c values were standardized to the Diabetes Control and Complications Trial (DCCT) reference (ref.) range of 4.05%−6.05% (20.7–42.6 mmol/mol) using the multiple of the mean transformation method to account for different laboratory methods [15]. Anthropometric measurements were performed in the local centers according to guidelines. The body‐mass index (BMI) is presented as standard deviation score (BMI‐SDS) according to German ref. data [16]. Immigrant background was defined as the child or one of the parents being born outside of Germany. Missing data on immigrant background were considered as a separate category in order to not exclude these individuals. The proportion with a confirmed immigrant background among all individuals and the proportion without information on immigrant status are given in the descriptive analysis. Postal codes of the residence at manifestation were used to assign the children to a degree of urbanization. Based on the population density of local administrative units as provided by Eurostat, three degrees of urbanization were defined: “urban areas” (at least 50% of the population living in an urban center with ≥1500 inhabitants/km^2^, and a minimum of 50,000 inhabitants collectively), “rural areas” (at least 50% of the population living in areas with <300 inhabitants/km^2^, and <5000 inhabitants collectively), and “suburban areas” collecting anything in between [17]. The physician density at the FS level in Germany is measured as number of inhabitants divided by the number of physicians based on data from the federal statistical office (DESTATIS) and the German medical association from the year 2022 [18]. The coverage of pediatricians is also provided by DESTATIS and the German medical association and is given as percentage of pediatricians per calculated number of required pediatricians per FS [10].

### 2.4. Statistical Analysis

**Figure 2:**
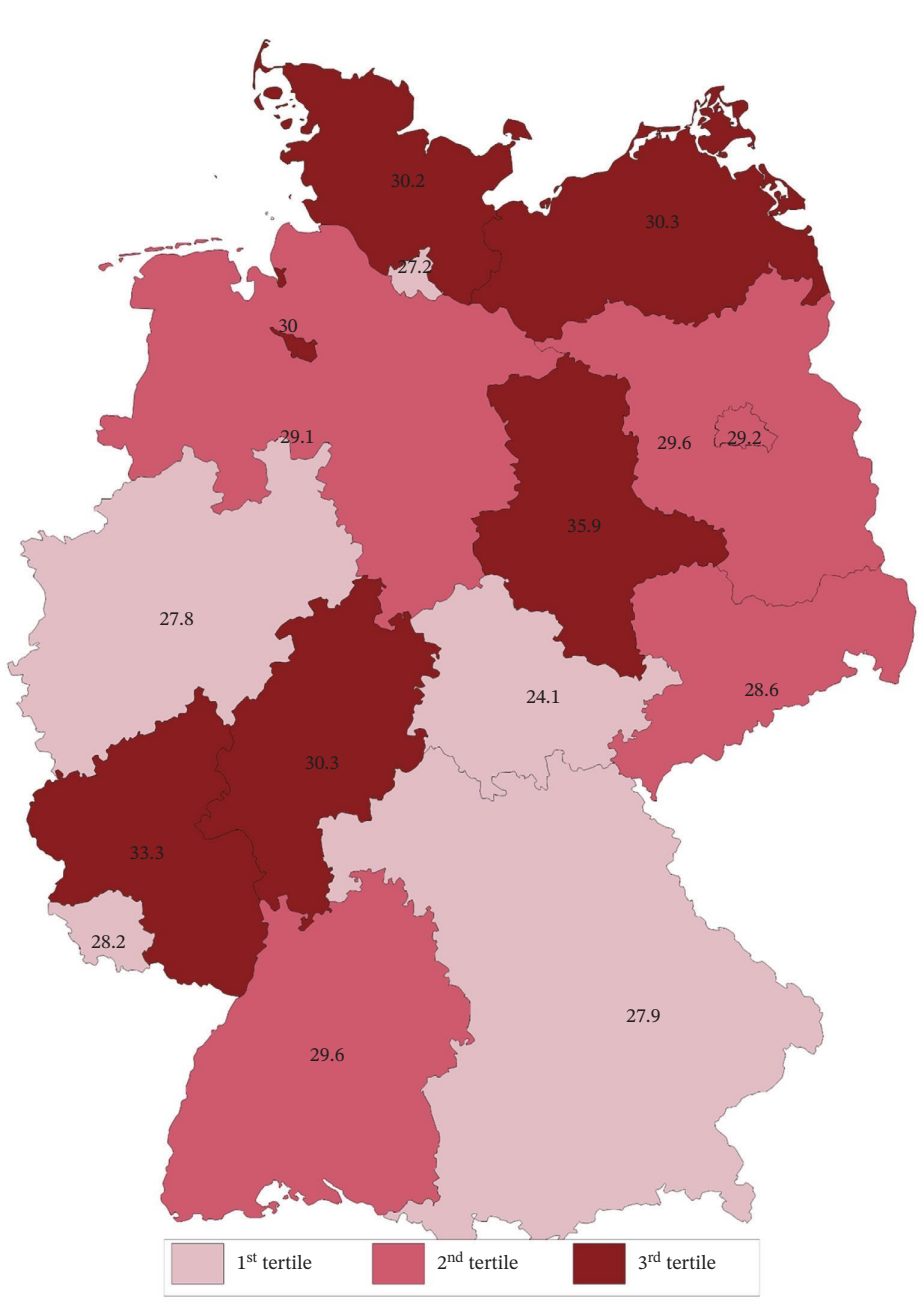
Map of all federal states in Germany with unadjusted DKA proportion at pediatric T1D onset and with color scheme by tertiles.

The results of descriptive statistics are shown as median with quartiles for continuous variables and as proportion for binary variables. *p*‐Values were calculated using the chi‐squared test for categorial variables and the Wilcoxon’s rank sum test for continuous variables and adjusted for multiple comparisons according to the Bonferroni–Holm [19] method. The raw proportion of DKA at T1D onset by FS in Germany is shown in Figure 2.

To analyze associations between individual demographic and postal code/FS level structural characteristics and the DKA proportion (dependent variable), a logistic regression model was implemented using sex, age at T1D onset (<3, 3 to <6, 6 to <9, 9 to <12, and 12 to <15 years), immigrant background, grouped years of diagnosis (2015–2017, 2018−2019, 2020–2021, and 2022−2023), urbanity index (urban, suburban, and rural areas), physician density, coverage of pediatric and adolescent physicians, and FSs with or without screening for T1D as independent variables. Additionally, a term for an interaction between physician density and coverage of pediatric and adolescent physicians was included. Based on this model, the relative risk (RR) for DKA was calculated for all predictors. For physician density the risk was calculated for a decrease of ten inhabitants per physician (higher physician density) and for the coverage of pediatric physicians the risk was calculated for an increase per 10% points (higher coverage). Therefore, for all predictors, RRs <1 for DKA indicate an advantageous association.

In a second step, the RR for DKA with regard to screening for T1D was calculated stratified for the sub‐categories of sex (ref. = female), age at T1D onset (ref. = 3 to <6 years), immigrant background (ref. = no immigrant background), year of diagnosis (ref. = 2015–2017), and urbanity index (ref. = urban), each adjusted for the other possible predictors.

These analyses were additionally conducted as sensitivity analysis with Bavaria only as FS with screening, excluding the FS Hamburg, Lower‐Saxony, and Saxony from this analysis. In order to provide more detailed evidence, we further investigated the group with the highest probability of being screened according to the Fr1da‐study. Hummel et al. [9] mention that, in Bavaria, until March 2019 only children aged 1.75–5.99 years were screened, after that, children between the ages of 1.75 and 10.99 years were offered screening. Given a median time‐lag of 22 months from screening to stage 3 T1D, most children who have been screened and developed T1D should be those within the onset‐years 2020–2023 aged 1.75–10.99 years from Bavaria [9]. Therefore, we compared those children with children of the same age and manifestation in the same years from FS without screening, again with exclusion of manifestations in Hamburg, Lower‐Saxony, and Saxony.

For all analyses a two‐sided *p*‐value of <0.05 was considered as significant. All statistical analyses were performed using statistical analysis software (SAS, SAS Institute Inc., Cary, NC, USA) Version 9.4.

## 3. Results

24,408 children and adolescents fulfilled the inclusion criteria. Median (with lower and upper quartile) age at T1D onset was 8.8 (5.3; 11.8) years, 54.3% were male, and 26.3% had an immigrant background. At clinical manifestation, median BMI‐SDS in the whole groups was −0.28 (−1.12; 0.53) and HbA1c was 11.1% (9.7; 12.8) or 98 mmol/mol (82; 116), respectively. Table 1 shows basic characteristics stratified by FS with and FS without screening availability. Figure 2 displays the raw proportion with DKA at T1D onset by FS from 2015 to 2023 in Germany. Most FSs revealed a DKA proportion of about 30%, the lowest DKA proportion was observed in Thuringia (24.1%), the highest proportions were observed in Rhineland‐Palatinate (33.3%) and in Saxony‐Anhalt (35.9%).

**Table 1:** Characteristics of the whole study cohort and stratified by federal state with/without availability of the Fr1da screening study.

Variable	*N*	All	Screening^a^ (*N* = 8068)	No screening^b^ (*N* = 16,340)	*p*‐Value
Sex (% male)	24,408	54.3	55.4	53.8	0.080
Age at T1D onset (years)	24,408	8.8 (5.3; 11.8)	8.7 (5.3; 11.8)	8.8 (5.4; 11.8)	0.653
Immigrant background (%)	24,408	26.3	22.8	28.0	<0.001
Unknown immigrant status	24,408	9.4	7.6	10.3	<0.001
BMI‐SDS	22,713	−0.3 (−1.1; 0.5)	−0.3 (−1.2; 0.5)	−0.3 (−1.1; 0.5)	0.136
HbA1c	22,842	—	—	—	—
%	—	11.1 (9.7; 12.8)	11.1 (9.6; 12.9)	11.1 (9.7; 12.7)	0.653
mmol/mol	—	98 (82; 116)	98 (81; 118)	98 (83; 116)	
With DKA (%)	24,408	29.1	28.4	29.4	0.332

*Note*: HbA1c, glycated hemoglobin. Screening, federal state in Germany that implemented the Fr1da screening program; no screening, federal state in Germany that did not implement the Fr1da screening study, regardless of other preventive or screening initiatives.

Abbreviations: BMI, body‐mass index; DKA, diabetic ketoacidosis; T1D, type 1 diabetes.

^a^Bavaria, Hamburg, Lower Saxony, and Saxony.

^b^All other federal states of Germany.

**Figure 3:**
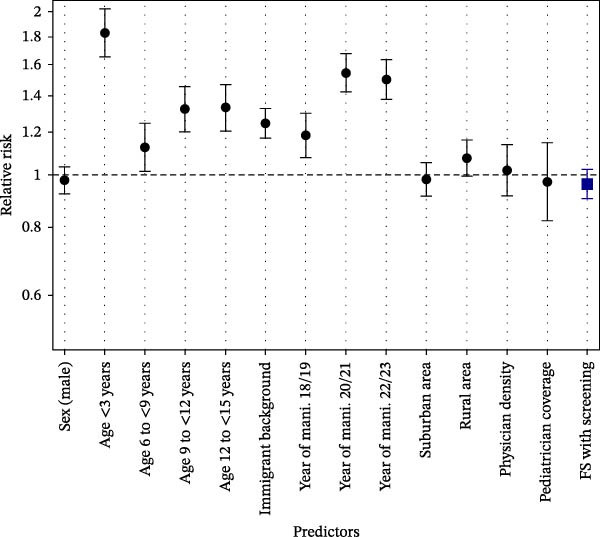
Relative risk for DKA at pediatric T1D onset dependent on demographic and context factors. Reference groups: sex: female; age: 3 to <6 years; no immigrant background; years of manifestation: 2015–2017; urban area; and federal state without screening. For physician density the risk was calculated for a decrease of ten inhabitants per physician and for the coverage of pediatric and adolescent physicians the risk was calculated for an increase per 10% points. FS, federal state; Mani, manifestation.

Among all predictors considered, early manifestation (below 3 years of age) revealed the highest RR (1.83, 95% CI [1.66–2.03], *p* < 0.001) for DKA, compared to the age group with the lowest rate of DKA (3 to <6 years). The next higher RR for DKA at T1D onset was observed for children and adolescents with manifestation during the years 2020–2021 (1.54 [1.42–1.67], *p* < 0.001) and 2022–2023 (1.49 [1.38–1.62], *p* < 0.001). Furthermore, immigrant background (1.28 [1.20–1.36], *p* < 0.001) and living in rural areas (1.09 [1.01–1.17], *p* = 0.037) revealed higher RRs for DKA (Figure 3). No significant association could be found regarding availability of screening for early‐stage T1D in the whole group (RR: 0.96 [0.93–1.03], *p* = 0.393) (Figure 3).

Stratified for subgroups, no significant association between FS with vs. without availability of screening could be found (Table 2). The lowest RRs were observed in the onset‐years 2015–2017 (RR: 0.91 [0.83–1.01], *p* = 0.063), the onset‐years 2022–2023 (RR: 0.93 [0.86–1.00], *p* = 0.066), and in urban areas (RR: 0.93 [0.86–1.00], *p* = 0.053) (Table 2).

**Table 2:** Relative risks (RRs) for DKA at T1D onset for individuals from federal states with^a^ vs. without screening^b^ availability for early stages of T1D in subgroups.

Subgroup	*N*	RR with 95% CI	*p*‐Value
Female	11,152	0.97 [0.91–1.03]	0.333
Male	13,256	0.96 [0.90–1.01]	0.122
Age at T1D onset: <3 years	2637	0.94 [0.86–1.04]	0.254
Age at T1D onset: 3 to <6 years	4496	0.96 [0.85–1.08]	0.481
Age at T1D onset: 6 to <9 years	5453	0.96 [0.87–1.06]	0.398
Age at T1D onset: 9 to <12 years	6237	0.99 [0.91–1.07]	0.768
Age at T1D onset: 12 to <15 years	5585	0.96 [0.88–1.04]	0.312
No immigrant background	15,704	0.98 [0.92–1.03]	0.356
With immigrant background	6409	0.95 [0.88–1.03]	0.190
Onset‐years 2015–2017	7064	0.91 [0.83–1.01]	0.063
Onset‐years 2018–2019	4995	1.01 [0.91–1.11]	0.875
Onset‐years 2020–2021	6422	0.98 [0.91–1.05]	0.581
Onset‐years 2022–2023	5927	0.93 [0.86–1.00]	0.066
Urban area	8510	0.93 [0.86–1.00]	0.053
Suburban area	8987	0.97 [0.90–1.05]	0.404
Rural area	6911	0.95 [0.88–1.03]	0.198

^a^Bavaria, Hamburg, Lower Saxony, and Saxony.

^b^All other federal states of Germany.

Sensitivity analysis with excluding Hamburg, Lower Saxony, and Saxony from the analysis, leaving only Bavaria as FS with screening availability produced similar results. The RR for DKA in Bavaria vs. FS without screening was 0.95 [0.87–1.04], *p* = 0.432. Again, no significant association regarding the RR for DKA in Bavaria vs. FS without the offer of screening excluding Hamburg, Lower Saxony, and Saxony could be detected in the sub‐group analysis. The lowest RRs were observed in children aged 6 to <9 years (RR: 0.89 [0.77–1.03], *p* = 0.117), in the onset‐years 2020–2021 (RR: 0.90 [0.81–1.00], *p* = 0.050), the onset‐years 2022–2023 (RR: 0.91 [0.82–1.02], *p* = 0.097), and in urban areas (RR: 0.92 [0.82–1.02], *p* = 0.125) (Table S1).

However, there was a significant risk reduction in the population with the highest possibility of being screened (children aged 1.75–10.99 years, with T1D onset in Bavaria from 2020 to 2023) with an estimated RR for DKA of 0.88 [0.80–0.97], *p* = 0.012 compared to children of the same age from FS without screening offer. Again, excluding Hamburg, Lower Saxony, and Saxony from the analysis. In contrast, in the same population of children aged 1.75–10.99 years with T1D onset in Bavaria, but for the years 2015–2019 there was no significant difference in the proportion with DKA compared to FS without screening offer (RR: 1.04 [0.91–1.19], *p* = 0.583).

## 4. Discussion

In Germany, the frequency of DKA in children and adolescents aged <15 years with a T1D onset between 2015 and 2023 was highest in the youngest children (<3 years) and higher during the COVID‐19 pandemic, in children with immigrant background, and in rural areas. Overall, no significant difference could be found regarding sex and physician density. In addition, no clear association between FSs that offer screening as part of a study for early stages of T1D and the proportion of DKA at population level could be found.

Even though there is evidence that screening for early stages of T1D is associated with a lower risk for delayed diabetes diagnosis with all its acute and long‐term complications in families participating in the Fr1da study [9, 20], it is difficult to assess its impact on the whole pediatric population in Germany.

Furthermore, other measures such as awareness campaigns in Stuttgart and in other countries were also reported to be associated with a reduced proportion of DKA [12, 21], but according to a meta‐analysis, only in one study the DKA proportion was lower than 10% [22]. Nevertheless, the overall DKA rate at pediatric T1D in Germany onset is still around 30%.

We could not show a significant association between children living in a FS where the Fr1da screening study is provided and the frequency of DKA at T1D manifestation on the population level by now. This was also true for the sensitivity analysis with Bavaria as only FS with screening availability, in the whole cohort, and in sub‐groups. However, we could find a significantly lower RR for DKA in the specific sub‐group with the highest probability of being screened (children aged 1.75–10.99 years with T1D onset in Bavaria from 2020 to 2023). This indicated that screening for early stages of T1D can possibly reduce the DKA‐rate, but to show an effect among the whole population it needs to be significantly expanded. This is also important taking recent results from other screening initiatives into account such as the Italian pilot screening program. Cherubini et al. [23] found lower OR for DKA in participating regions, especially severe DKA was markedly lower (OR: 0.51, 95% CI: 0.39–0.67, *p* < 0.001) than in regions not participating in the screening program). However, the Italian group did not find significant differences between 2023, the year with preparatory measures for the screening project and 2024, when screening was implemented. This suggests that the awareness of the screening project itself may have led to a reduction in DKA at T1D onset, even before the screening project had an impact. The authors also mention the important role of pediatricians in implementing the screening and also in raising awareness of the symptoms of early T1D among themselves and families. With regard to the still regional study approach in Germany, the following should be noted:

Since the local start of screening in Bavaria in February 2015 and the expansion to Saxony, Lower Saxony, and Hamburg by 2021, 188,842 children aged 2–11 years have participated in the German Fr1da study [24]. Due to the slow development of screening numbers, a change in the DKA rate at clinical onset cannot be expected for Germany at this time. Assuming 3200 manifestations of clinical T1D in children and adolescents aged <18 years annually in Germany with a rate of 30% (*n* = 960) presenting with DKA at diagnosis [2, 24, 25], a reduction of DKA rate to 27% (*n* = 864; according to a 10% relative reduction) would be detectable with a power of 84% at the population level. In order to achieve this 10% relative reduction and on the basis of the reported DKA rate of 2.5% in this group [9, 24], approximately 350 annual cases with newly diagnosed clinical T1D must occur in children with detected early‐stage T1D. Following the screening framework and assumptions according to Bonifacio et al. [24], this figure would be achieved after annual population screening in children at ages 2 and 6 years (each approximately 800,000 children per year) for 11 years with an approximate screening rate of 20% (corresponding to a total of approximately 2.88 million children screened).

Furthermore, it has to be considered that screening has so far only been carried out on a voluntary basis and is accompanied by high workload for the pediatricians which makes it difficult to achieve a high coverage. Therefore, it cannot be ruled out that there is a selection bias: participants in research studies are often more educated, have higher income, live in geographic areas with a higher socioeconomic status [26], and are more interested in medical topics with different use of health care [27]. One might argue that participants of the Fr1da study, therefore, have generally a lower pre‐test probability of developing DKA at T1D onset. However, only a capillary blood draw is necessary in order to take part which can be done by the primary pediatrician, so the inhibition threshold should be quite low. Further, it could be shown that around 20% of Fr1da participants are from households with low‐income and the proportion from urban (Fr1da: 34%, DPV: 35%), suburban (Fr1da: 38%, DPV: 37%), and rural areas (Fr1da: 28%, DPV: 28%) is similarly distributed in the Fr1da study and in the whole DPV cohort [28].

One issue for some families that needs to be addressed is the psychological burden and the fear of knowing that the child has autoantibodies and therefore is at risk of developing T1D in the future without knowing the exact time point [29]. It can take many years until the disease manifests, as less than half of the children with detected stage 1 T1D manifest within 5 years [30, 31]. However, there is also evidence that families who have participated in a screening study show less psychological distress [8] and better diabetes‐related quality of life due to the supervision and support [32]. Although T1D is a chronic disease with no cure, new immunomodulatory drugs, such as teplizumab, can preserve beta‐cells and delay T1D onset [33]. Teplizumab is currently approved by the US Food and Drug Administration (FDA) [34] but not by the European Medicines Agency (EMA). In November 2024, Teplizumab was included in the compassionate use program of the German Paul‐Ehrlich‐Institut [35].

A small group (about 7% in Germany) of children develop T1D without detectable autoantibodies at onset of symptoms [36]. For these children an autoantibody‐based screening might not be effective. Otherwise, it has been shown that some patients develop diabetes specific antibodies initially and lose them before manifestation [37]. These children could still be picked up by a screening study.

An important finding of our study is that infants under 3 years of age have the highest risk of developing DKA with T1D onset, while the average age of seroconversion to multiple islet autoantibodies is 2–3 years of age [38]. DKA proportion is lower in those with a familial history of T1D [39], indicating that increased awareness for T1D could reduce the risk for DKA at manifestation. This might be especially important for very young children whose clinical symptoms are often difficult to interpret. The dilemma is that earlier screening for antibodies results in a higher proportion of false‐negative, and the later autoantibodies are detected, the more young infants cannot be protected from DKA despite screening. To overcome these difficulties, consecutive screening at two different time points has been proposed and is already being offered to children participating in the Fr1da study [24]. A combination of the Freder1k‐study, which was launched in Dresden in 2016 to determine genetic risk using a polygenic risk score in newborns [40] and the Fr1da study may be a promising approach to sensitize families to T1D at an early stage [41]. To achieve the greatest possible success, the studies would have to be offered nationwide.

We considered several other structural and demographic aspects that might interact with screening initiatives and in part are known to be associated with DKA at T1D onset. Higher DKA proportions among children with immigrant background have been detected in previous reports from the German DPV‐registry [42]. Language barriers complicate medical information and education. Furthermore, the DPV initiative and cohorts from other countries found that DKA at T1D onset is associated with other structural factors such as socioeconomic deprivation [43] or the urbanity index [44]. Other structural aspects such as physician density at a state level did not seem to have any effect on the DKA proportion. We also considered the year of manifestation to account for the known impact of the COVID‐19 pandemic on the DKA‐rate at T1D onset [1, 2, 45].

## 5. Strengths and Limitations

The strength of this study is the high number of documented children with T1D in Germany with available data at clinical onset. The major limitation is that no person‐related information on the participation in the screening study was available, and screening did not start at the same time parallel in all four participating FSs. However, the possibility and likelihood for children being screened varies in terms of residence, age, year of onset, and social status. Furthermore, there are other screening initiatives in Germany, such as the Freder1k‐study, in which newborns are screened based on polygenetic risk score, but most children screened did not yet develop diabetes and the impact on the population based DKA rate is, therefore, questionable. For this reason, we focused only on the Fr1da initiative in this study and conducted sensitivity analyses for Bavaria only. Other approaches, such as the awareness campaign in Stuttgart (Baden‐Württemberg), complicated the analysis since these other measures to reduce the frequency of DKA at pediatric T1D onset might interact with the screening initiatives.

## 6. Conclusion

We could not find clear evidence that screening for T1D was associated with lower DKA risk at the population level so far, but in the sub‐group of Bavarian children who had the highest probability of being screened, the RR for DKA was significantly lower compared to other FSs in Germany. Screening for early stages of T1D can be a useful tool to prevent DKA at onset, but it will need large efforts to achieve a high enough coverage in order to significantly reduce the DKA rate at the population level. It seems likely that this can only be reached by a standardized German screening program which is routinely offered to every family. Furthermore, an antibody‐based screening alone will not be sufficient to identify and protect very young children which are particularly at risk for DKA at T1D onset and the impact of screening on children with a long interval between screening and onset of clinical disease needs to be investigated, as awareness may decline. Supporting strategies are important to be implemented besides screening initiatives and potential adverse psychological effects of screening as well as cost efficiency and medical resources for screening and follow‐up of at‐risk‐children need to be considered.

## Author Contributions

Alexander J. Eckert analyzed the data, wrote the first draft, and revised the paper after the critical review of all co‐authors. Alexander J. Eckert and Reinhard W. Holl conceived and coordinated the study. Joachim Rosenbauer, Clemens Kamrath, Marina Sindichakis, Ansgar Thimm, Martin Holder, Jantje Weiskorn, Daniel P. Lorenz, Susanne Gonzalves, Gita Gemulla, Susanne Gonzalves, Ute Ohlenschläger, Dieter Hüseman, Katja Palm, Andreas Lemmer, Valentina Lahn, Antonia Müller, Donald Wurm, Marjatta Wütherich, and Reinhard W. Holl participated in the DPV initiative by providing data and reviewed the article critically.

## Funding

This study was supported by the German Federal Ministry for Education and Research within the German Centre for Diabetes Research, DZD (Grant 82DZD14H03), the German Robert Koch Institute (RKI), and the German Diabetes Association (DDG). Open Access funding enabled and organized by Projekt DEAL.

## Disclosure

The sponsors were not involved in data acquisition or analysis.

## Conflicts of Interest

Antonia Müller received a lecture fee from Sanofi. Jantje Weiskorn received lecture fees from Sanofi. Clemens Kamrath received lecture fees from Sanofi. The other authors declare no conflicts of interest.

## Supporting Information

Additional supporting information can be found online in the Supporting Information section.

## Supporting information


**Supporting Information** Table S1. Relative risks (RRs) for DKA at T1D onset for individuals from Bavaria (screening study) vs. FS without screening availability for early stages of T1D in subgroups, excluding Hamburg, Lower Saxony, and Saxony.

## Data Availability

The data that support the findings of this study are available upon request from the corresponding author. The data are not publicly available due to privacy or ethical restrictions.
