# Supplementary material for: Is the Current Screening Availability for Early Stages of Type 1 Diabetes in Germany Related to the Population‐Based Frequency of Diabetic Ketoacidosis at Clinical Manifestation in Children and Adolescents
Source: Pediatr Diabetes. 2026 Feb 8;2026:6905472. doi: 10.1155/pedi/6905472 (PMC12884009; doi:10.1155/pedi/6905472)
Supplement: Supplementary file 1 — Supporting Information Table S1. Relative risks (RRs) for DKA at T1D onset for individuals from Bavaria (screening study) vs. FS without screening availability for early stages of T1D in subgroups, excluding Hamburg, Lower Saxony, and Saxony. [file PEDI-2026-6905472-s001.docx]

**Supplementary material**

**Supplementary table 1. Relative risks (RR) for DKA at T1D onset for individuals from Bavaria (screening study) vs. FS without screening availability for early stages of T1D in subgroups, excluding Hamburg, Lower Saxony and Saxony.**

| **Subgroup** | **N** | **RR with 95%-CI** | **p** |
| --- | --- | --- | --- |
| **Female** | 9,101 | 0.96 [0.88-1.05] | 0.374 |
| **Male** | 10,685 | 0.93 [0.86-1.01] | 0.096 |
| **Age at T1D onset: <3 years** | 2,161 | 0.97 [0.85-1.11] | 0.669 |
| **Age at T1D onset: 3-<6 years** | 3,641 | 0.92 [0.78-1.09] | 0.333 |
| **Age at T1D onset: 6-<9 years** | 4,434 | 0.89 [0.77-1.03] | 0.117 |
| **Age at T1D onset: 9-<12 years** | 5,063 | 0.98 [0.87-1.11] | 0.765 |
| **Age at T1D onset: 12-<15 years** | 4,487 | 0.95 [0.84-1.07] | 0.401 |
| **No immigrant background** | 12,205 | 1.00 [0.92-1.08] | 0.916 |
| **With immigrant background** | 5,438 | 0.90 [0.81-1.00] | 0.052 |
| **Onset-years 2015-2017** | 5,727 | 0.98 [0.85-1.12] | 0.739 |
| **Onset-years 2018-2019** | 4,005 | 1.04 [0.90-1.20] | 0.592 |
| **Onset-years 2020-2021** | 5,223 | 0.90 [0.81-1.00] | 0.050 |
| **Onset-years 2022-2023** | 4,831 | 0.91 [0.82-1.02] | 0.097 |
| **Urban area** | 6,930 | 0.92 [0.82-1.02] | 0.125 |
| **Suburban area** | 7,623 | 0.98 [0.88-1.10] | 0.791 |
| **Rural area** | 5,233 | 0.98 [0.88-1.10] | 0.743 |
